# Phosphide of Zinc

**Published:** 1875-12

**Authors:** 


Phosphide of Zinc.—Nervous exhaustion or general nervous debility is usually associated with functional derangement in some one of the important organs. The patient is irritable, has feelings of alarm, confusion of the mental faculties, uncertainty of memory, wakeful nights, a general feeling of weakness, and although there is no wasting of muscle there is a marked weakness of the spinal muscles as indicated by the leaning and stooping posture of the patient, and tottering gait.

In this condition we have great nervous and mental depressions, pain and weariness through the body. Under the influence of the zinc phosphide the appetite is increased, the digestion is improved, and nutrition begins to show its effects upon the muscles, brain and nerves. Accordingly we find the patients who were so feeble that even a short ride or walk occasioned fatigue, and who were signally deficient both in the will and capacity for exertion, soon begin to develop under the treatment, an activity and vigor that is sometimes surprising.

Zinc phosphide can be combined with other drugs, with good advantage, excepting acids.

The following pill I have found invaluable in chorea:

| | |
|---|---|
| ℞.—Phosphide of Zinc, . . . | gr. x. |
| Alc. Ext. Cimicifuga, . . . | gr. xxv. |
| " " Hyoscyami, . . . | gr. xx. |

M. ft. pil. no. L.

Sig. One to two pills three times a day, for a child twelve years of age.

*Spinal Irritation.*—As the faculties of the spinal cord are built up by organization so must they be kept up by due nutrition. If not so preserved in vigor—if exhausted by any means ill effects are manifest in degenerate action, therefore spinal irritation is in fact a mal-nutrition.

The differential diagnosis between spinal irritation and spinal congestion or inflammation is one, purely of degree and effect.

The following pill has proved curative in a number of very stubborn cases.

| | |
|---|---|
| ℞.—Phosphide of Zinc, . . . | gr. x. |
| Phosphate of Iron, . . . | gr. lxxx. |
| Quinine, . . . . | gr. xl. |
| Alc. Ext. Hyoscyami, . . . | gr. xxx. |
| " " Belladonna, . . | gr. xii. |

M. ft. pil. no. L, and sugar-coat them.

Sig. One pill every four hours.

